# Mechanical properties of charcoal and its representativeness of vegetation in northern China

**DOI:** 10.1371/journal.pone.0267044

**Published:** 2022-04-14

**Authors:** Nan Sun, Xiabo Li, Fan Luo, Liang Xiao

**Affiliations:** 1 School of Earth Science and Resources, Chang’an University, Xi’an, China; 2 Shaanxi Key Laboratory of Early Life and Environments, Northwest University, Xi’an, Shaanxi, China; Institute of Earth and Environment, Chinese Academy of Sciences, CHINA

## Abstract

The objective of this paper is to examine the representativeness of charcoal taxa at archeological sites in northern China. We carried out standardized laboratory compression tests on 168 samples representing 21 taxa charred at four different temperatures to characterize the mechanical properties of common taxa in temperate China. The results indicate that significant fragmentation differences occur between taxa. Ring-porous/semi-ring-porous taxa with a moderate density (>0.55 g/cm^3^) are overrepresented, while those with a very low to low density (<0.55 g/cm^3^) are moderately represented. Diffuse-porous taxa with slightly dense uniseriate rays, rare multiseriate rays and distinct helical thickenings are underrepresented, and those with slightly dense multiseriate rays are overrepresented, while those with rare to moderate multiseriate rays and helical thickening absence are moderately represented. Gymnosperm trees are generally well represented. Among the ubiquitous taxa at the archeological sites across northern China, *Quercus* and *Ulmus* may be overrepresented, and *Pinus*, *Salix*, *Populus*, and *Acer* may be underrepresented, while *Betula* may be moderately represented.

## 1. Introduction

Wood has widely been used as a resource by humans for a long time. Charcoal, as a product of incomplete wood combustion [1], is an effective indicator revealing fire activity during prehistorical and historical periods [2–5]. Relatively large pieces of charcoal retain structural features [6, 7], allowing identification of taxa with the help of modern reference collections and identification manuals/databases. To obtain a better understanding of charcoal assemblages in strata, Chabal (1997) has proposed that the number and percentage of charcoal fragments of different taxa could be used to estimate the occurrence in primeval vegetation [8]. This method has become an important approach to reconstruct paleovegetation and paleoenvironment conditions [9–12], to reconstruct the paleoclimate [13–15] and to examine early human activities [15–31]. However, the application of this statistical method assumes that the charcoal of all taxa exhibit similar crushing characteristics [8, 32, 33].

Studies have demonstrated that taxa differences in the number of charcoal fragments occur during the carbonization process [34]. Charcoal is more brittle than wood [35]. When charcoal is buried in soil for a long time, it becomes further fragmented via pressure of the overlying strata, humidity and grain size of the soils, movements of the sediments, etc. [36]. In this paper, we study the impact of the pressure of the overlying strata on charcoal. Owing to the distinct physical and chemical properties of different taxa of wood, notable differences in the compressive resistance of the formed charcoal are exhibited [36, 37]. Under pressure, certain taxa of charcoal easily break to form numerous fragments, while others do not easily break and thus form relatively few fragments. Therefore, quantification of the proportion of only charcoal fragments of different taxa for paleovegetation restoration may cause errors in the determination of the vegetation type. Hence, identification of over- and underrepresented taxa facilitates improvement in the interpretation accuracy of charcoal assemblages.

A large amount of charcoal is often preserved at archeological sites, which have become the main location from which to obtain charcoal. The distribution of archeological sites in China is generally bound to the Tengchong-Heihe line, with more sites in the east than in the west and more sites in the north than in the south, which indicates a pattern of diminishing surroundings with the Yangtze and Yellow River basins as centers [38]. Therefore, charcoal representativeness research has mainly been conducted at the archeological sites in northern China. Statistical analysis of published charcoal taxa reports targeting archeological sites in northern China shows that *Betula Pinus*, *Quercus*, *Salix*, and *Ulmus* are ubiquitous taxa [10, 14, 19–26]. *Betula*, *Pinus* and *Quercus* occur at many of the above archeological sites and often are the dominant taxa [10, 14, 19–20, 23]. Whether the proportion of the charcoal content of these taxa is consistent with the proportion of the paleovegetation composition is a key issue that we must resolve to accurately understand the vegetation type.

The distribution of vegetation in China exhibits obvious zonal regularity. The north is dominated by temperate deciduous broad-leaved forests, and this vegetation belt is also a high-occurrence area of archeological sites. This article combines the charcoal taxa commonly found at archeological sites and modern tree taxa commonly occurring in northern China. A total of 21 taxa, including *Acer*, *Betula*, *Cinnamomum*, *Cotinus*, *Cunninghamia*, *Diospyros*, *Ginkgo*, *Magnolia*, *Metasequoia*, *Padus*, *Paulownia*, *Photinia*, *Picrasma*, *Pinus*, *Populus*, *Pteroceltis*, *Quercus*, *Salix*, *Tilia*, *Toxicodendron* and *Ulmus*, is studied via charring and compression experiments. Then, we analyze the changes in samples before and after the experiments and identify possible over- and underrepresented taxa to improve the accuracy of future paleovegetation restoration.

## 2. Materials and methods

### 2.1. Sample preparation

**Fig 1:**
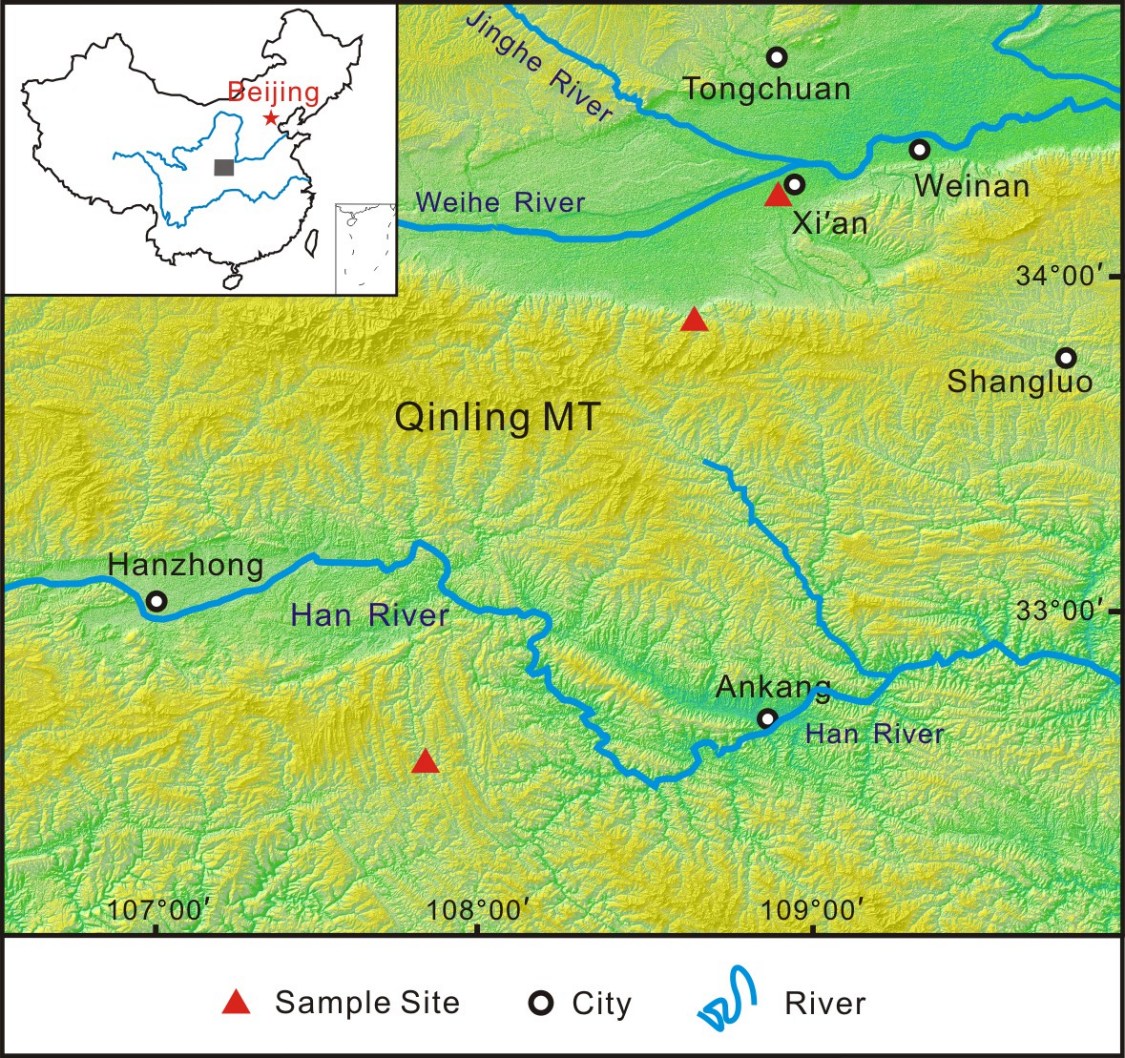
Location of sampling sites.

Wood samples of various modern tree taxa were collected at three sites in Xi’an, the Qinling Mountains and Hanzhong (Fig 1). Considering that most of the firewood collected by our ancestors were twigs, all samples were retrieved from branches with a diameter of approximately 3 cm, dried naturally for three weeks, and then placed in a 1.5 cm×1.5 cm×4 cm cuboid. We prepared a total of 168 samples representing 21 taxa, with 8 identical samples for each taxa.

### 2.2. Charring experiments

Since the natural combustion temperature generally ranges from 300–600°C [39], the charcoal encountered at archeological sites more likely represents wood charred at relatively low temperatures (for example, wood buried in ash and therefore not directly exposed to flames) or wood removed from fireplaces before the end of the burning process [35, 36]. Therefore, the chosen experimental temperatures were 300°C, 400°C and 500°C. Pieces of wood were individually wrapped in aluminum foil and charred in a muffle furnace following established protocols at the above three temperatures. Moreover, to better represent the historic process of fire use, we also conducted wildfire experiments (WF) under open conditions. Two parallel samples of each taxa were selected for the experiments under the above four sets of conditions.

In the muffle furnace, the temperature was quickly raised to the specified temperature, and the sample was then heated for 30 minutes. In the wildfire experiment, dry firewood was employed as fuel. Samples were also individually wrapped in aluminum foil and heated for 30 min in an outdoor open environment to simulate the fire used by early human. After charring, their volume, weight, and density were measured.

### 2.3. Compression experiments

**Fig 2:**
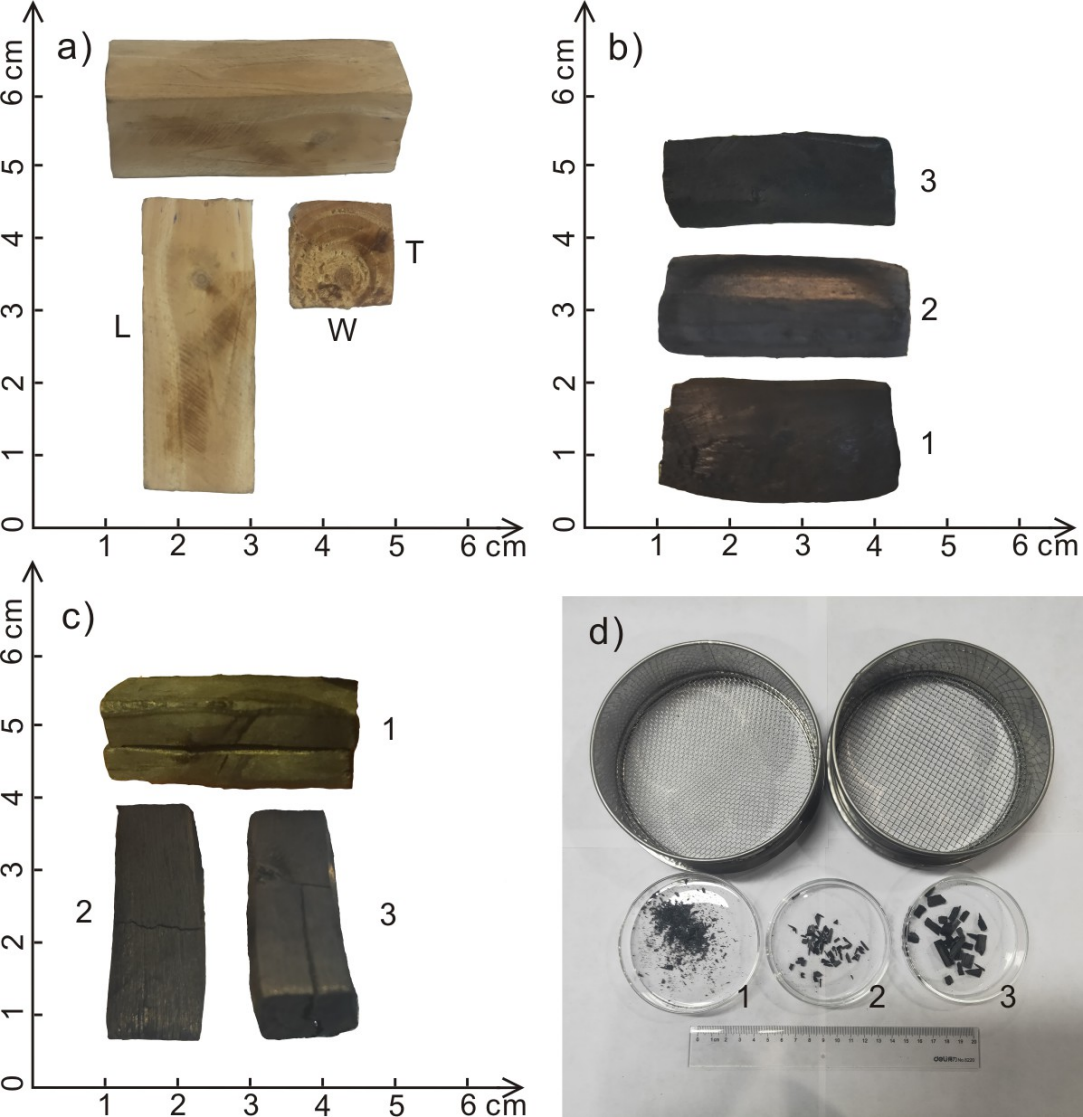
(a) Wood sample (L: length, W: width, T: thickness); (b) charcoal volume at 400°C (1. *Cunninghamia*, 2. *Cinnamomum*, 3. *Magnolia*); (c) part of the charcoal fissures at 500°C (1. *Cotinus*, 2. *Betula*, 3. *Quercus*); (d) charcoal fragments after compression test (1.<2mm, 2.2-4mm, 3.>4mm).

When charcoal is buried in soil, it will be crushed via compression due to overlying strata. Therefore, we adopted a standard compression testing machine (Landmark 370.10) to perform compression experiments on charcoal samples to simulate the pressure exerted by overlying formations, the experiments were performed in the open laboratory of Chang’an University [37, 40]. Compression was applied along the radial longitudinal direction (perpendicular to the fibers), and the descending speed was set to 2 mm/min for 30s from the time of contact with the sample, i.e., the descending displacement was 2.5 mm (the charcoal height was approximately 12–13 mm). The samples were placed in a plastic sealed bag so that the fragments could be recovered at the end of the experiment. Finally, 2- and 4-mm filter screens were employed to filter the sample fragments, and the number of charcoal fragments 2–4 mm and larger than 4 mm in size was counted (Fig 2D).

### 2.4. Flotation experiments

As the charcoal in the soil at archeological sites is mainly obtained via flotation [41], to evaluate the impact of this method on the degree of charcoal fragmentation, we placed the samples charred under wildfire conditions in a bucket with water and soil sediment after the compression experiment and stirred them clockwise, at a speed of approximately 3–4 revolutions per second. After stirring for approximately 10 s, the number of charcoal fragments was again determined via counting.

## 3. Results

### 3.1. Physical form changes

**Fig 3:**
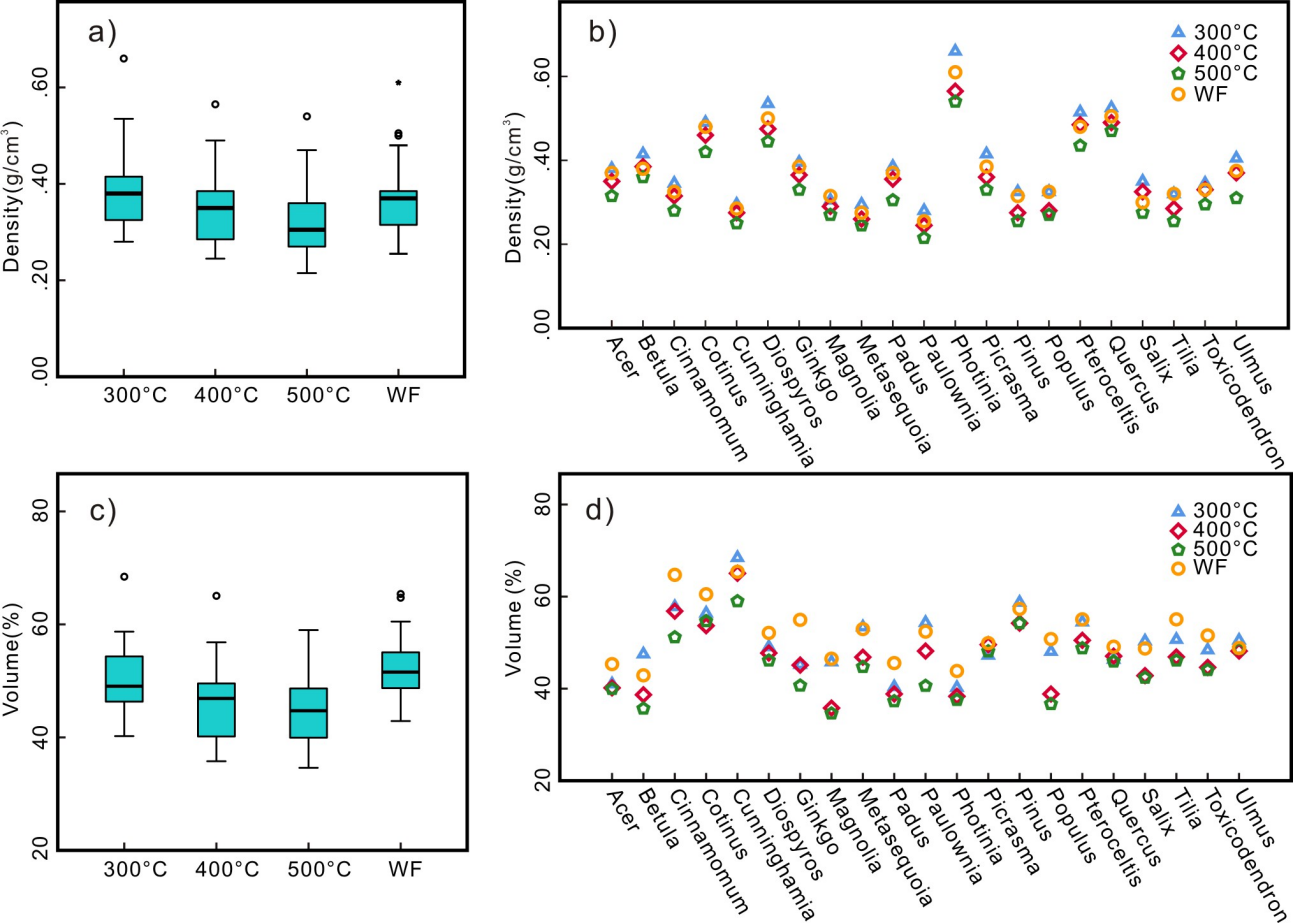
Charcoal density after carbonization (a) different temperatures; (b) different taxa; Volume loss after carbonization (c) different temperatures; (d) different taxa.

After wood carbonization, due to the loss of volatile matter, the mass, volume, and density decrease to varying degrees, and with increasing temperature, these parameters exhibit an overall decreasing tendency (S1 Table and Fig 3A and 3C). During this process, the charcoal of the different taxa exhibited obvious differences. The charcoal density is directly determined by the density of the original wood [37], so the charcoal density of the different taxa revealed notable differences (Fig 3B). Under the various temperature conditions, *Photinia* attained the highest density (the density at 300°C, 400°C and 500°C and under field conditions was 0.66, 0.57, 0.54 and 0.61 g/cm^3^, respectively). The density of the *Paulownia* charcoal was the lowest (the density at 300°C, 400°C and 500°C and under field conditions was 0.28, 0.24, 0.22 and 0.26 g/cm^3^, respectively). The same initial volume of wood leads to different volumes of charcoal, as different taxa shrink in varying degrees. Among them, *Magnolia* experienced the largest volume loss (a volume loss of 54%, 63%, 65% and 53% at 300°C, 400°C, 500°C and field conditions, respectively), and *Cunninghamia* experienced the smallest volume loss (a volume loss of 32%, 35%, 41% and 35% at 300°C, 400°C, 500°C and field conditions, respectively) (Figs 2B and 3D).

In addition, during carbonization, with increasing temperature, the charcoal integrity deteriorated. At 300°C, all charcoal samples remained relatively intact, and no obvious fissures were formed. At 400°C, 1–3 fissures were formed in *Betula*, *Diospyros*, *Magnolia*, *Metasequoia*, *Photinia* and *Ulmus* and among which the first four broke, producing 2–3 fragments. At 500°C, most charcoal samples exhibited 2–4 fissures (Fig 2C). Among them, *Betula*, *Cotinus*, *Diospyros*, *Ginkgo*, *Magnolia*, *Photinia*, *Populus*, *Prunus*, *Salix* and *Ulmus* broke, resulting in 2–4 fragments. In the wildfire environment, most charcoal samples also produced 2–4 fissures, among which *Diospyros*, *Padus*, *Paulownia*, *Photinia*, *Prunus*, *Salix*, *Tilia* and *Ulmus* cracked, resulting in 2–4 pieces. Certain tree taxa, such as *Acer*, *Picrasma*, *Pinus* and *Toxicodendron*, exhibited no fissures or breaks at all temperatures (S1 Table).

### 3.2. Fragmentation

**Fig 4:**
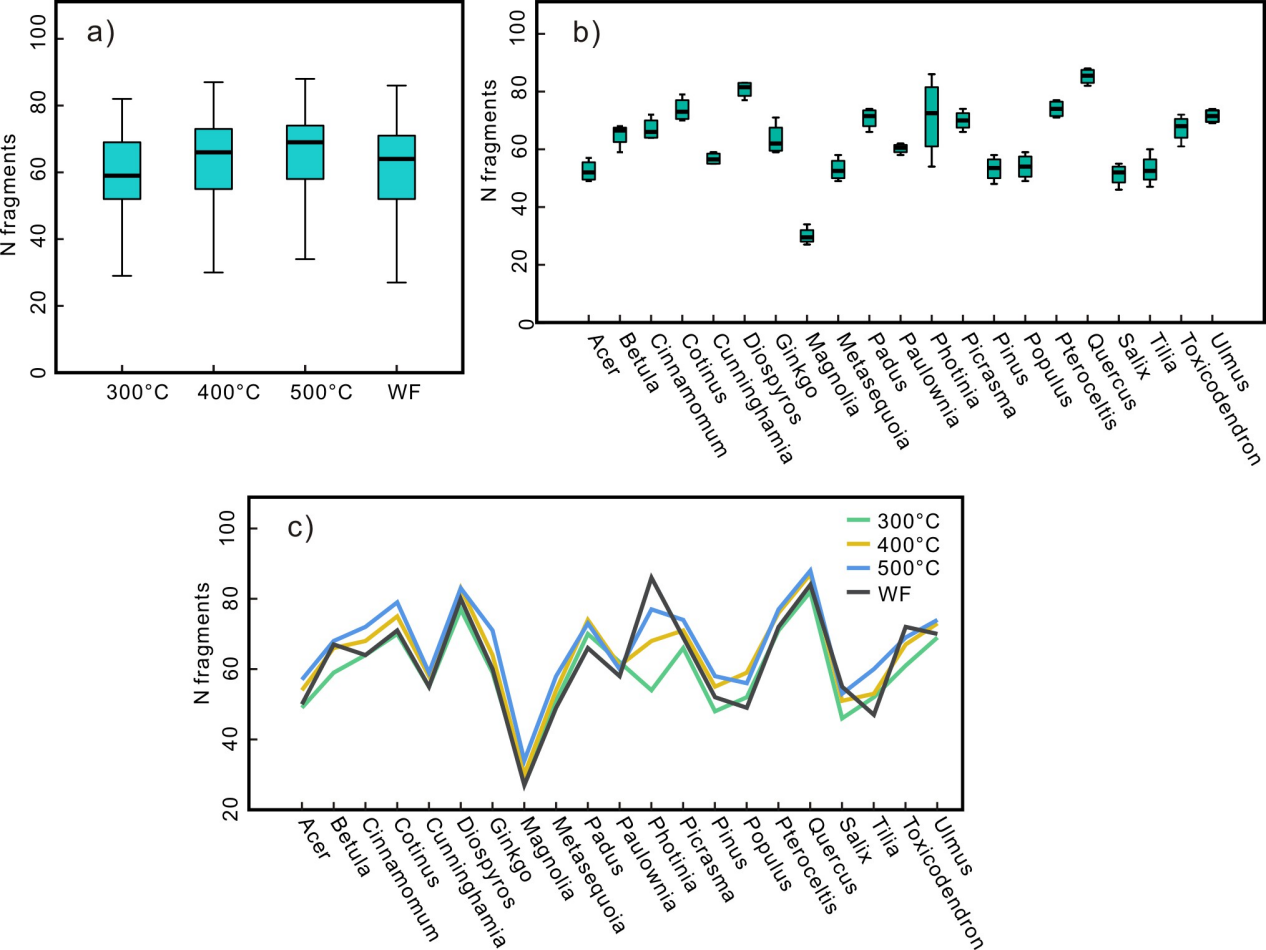
The number of charcoal fragments after the pressure experiment (a) at different temperatures; (b) different taxa (c) line chart of changes in the number of fragments at different temperatures.

The number of charcoal fragments after compression exhibited an increasing trend with increasing temperature (Fig 4A). The samples produced approximately 59, 64, 67, and 60 fragments on average at 300°C, 400°C and 500°C and under wildfire conditions, respectively. There were notable differences in the number of charcoal fragments among the different taxa (Fig 4B). Among them, the number of charcoal fragments in *Quercus* and *Diospyros* was the largest, exceeding the average value by 30% and 28%, respectively. *Cotinus*, *Padus*, *Photinia* and *Pteroceltis* exceeded the average value by approximately 15%, while *Picrasma* and *Ulmus* exceeded the average value by approximately 10%. *Magnolia* produced the least fragments, with only approximately half of the average value. *Acer*, *Metasequoia*, *Pinus*, *Populus*, *Salix* and *Tilia* produced approximately 15% less fragments than the average value, and the number of fragments of *Cunninghamia* was approximately 10% below the average value. The number of fragments of *Betula*, *Cinnamomum*, *Ginkgo*, *Paulownia* and *Toxicodendron* was close to the average value (Fig 4B). This trend basically remained the same under all temperature conditions (Fig 4C).

**Fig 5:**
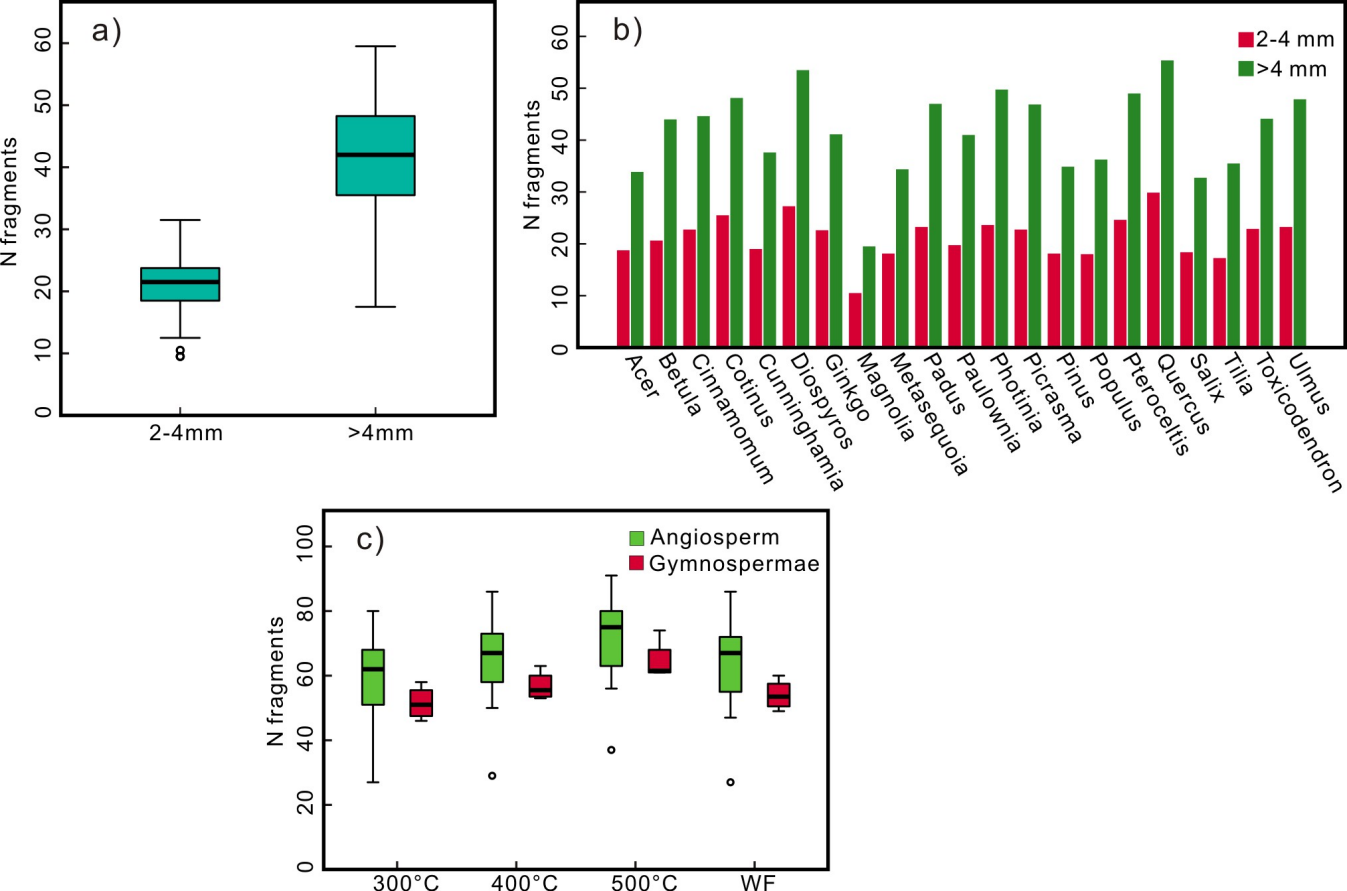
(a) Average number of fragments of different sizes; (b) number of fragments of different sizes in different taxa; (c) number of charcoal fragments of gymnosperm and angiosperm.

By counting the number of fragments 2–4 mm and >4 mm in size, the ratio of the number of fragments of these two sizes was found to be approximately 1/2 (Fig 5A), and the ratio between the various taxa was very similar (Fig 5B). This indicates that the crushing modes of charcoal are basically similar when broken into fragments of different sizes. Therefore, counting the number of charcoal fragments larger than 4 mm for the various taxa could suitably reflect the difference in the degree of fragmentation.

### 3.3. Influence of the flotation method on the number of charcoal fragments

After the compression experiment, the charcoal fragments prepared in the wildfire environment were stirred, and the number of fragments was again determined after stirring. The results indicated that although stirring the charcoal fragments in water slightly increased the number of fragments larger than 2 mm, this increase did not exhibit obvious differences among the various taxa (S1 Table). The flotation method does not cause obvious deviations in the charcoal statistics.

## 4. Discussion

### 4.1. Effect of wood carbonization on the degree of charcoal fragmentation

During the carbonization of wood, increasing carbonization temperature led to increasing brittleness in charcoal [35], resulting in an increase in the number of cracks and fragments in charcoal (S1 Table). One-way analysis of variance (ANOVA) revealed a significant correlation between the number of fragments and temperature (Table 1). Thus, increasing the temperature in the carbonization process caused the resultant charcoal to break more easily. However, in this process, some taxa produced only cracks without breaking, and other taxa broke into multiple pieces. (S1 Table), indicates that the carbonization process caused inter-taxa differences in the number of charcoal fragments.

**Table 1:** Effect of fragments and fissures from charring experimenton on temperature (ANOVA).

Variable	DDL	Sum of the squares	F value	p-Value
fissures	3	12.73	10.075	<0.001
fragments	3	9.56	4.019	0.010

### 4.2. Impact of the wood physical and chemical properties on the degree of charcoal breakage

After the compression experiment, the charcoal broke further, and the number of charcoal fragments varied greatly among the different taxa. This difference exhibited a good consistency under the various temperature conditions., i.e., the charcoal taxa that produced more (or fewer) fragments also produced more (or fewer) fragments under the various temperature conditions, and the proportion of charcoal fragments for the different taxa basically remained similar at the different temperatures (Fig 4C). One-way ANOVA results showed that there was a significant correlation between the charcoal taxa and number of fragments (p<0.001) (Table 2).

**Table 2:** Effect of taxa, temperature, and the number of fragments and fissures after in charring experimenton on fragmentation in compression experiment (ANOVA).

Variable	DDL	Sum of the squares	F value	p-Value
taxa	20	12376.73	16.532	<0.001
Temperature	2	1414.32	4.535	0.15
fragments	4	1510.44	1.782	0.126
fissures	5	1969.55	2.407	0.044

The main components of biomass are lignin, cellulose and hemicellulose. During wood carbonization, these three components sequentially undergo pyrolysis. Hemicellulose is basically completely pyrolyzed at 300°C and cellulose at 400°C. In contrast, lignin pyrolysis occurs in a wide temperature range (160~900°C) [42], and the mass loss does not exceed 60% even if the temperature reaches 600°C [43]. Therefore, the content of lignin may affect the compressive strength of charcoal [44, 45].

Generally, the lignin content of gymnosperms ranges from 25% to 35%, and the lignin content of angiosperms ranges from 20–25% [46]. Gymnosperms typically have a higher lignin content than angiosperms. The number of charcoal fragments produced by the four gymnosperms (*Cunninghamia*, *Ginkgo*, *Metasequoia*, and *Pinus*) in this study are all within the range of variation in the number of charcoal fragments produced by the angiosperms (Figs 4B and 5C). Therefore, we propose that the chemical composition imposes little effect on the differences in mechanical properties between taxa.

**Fig 6:**
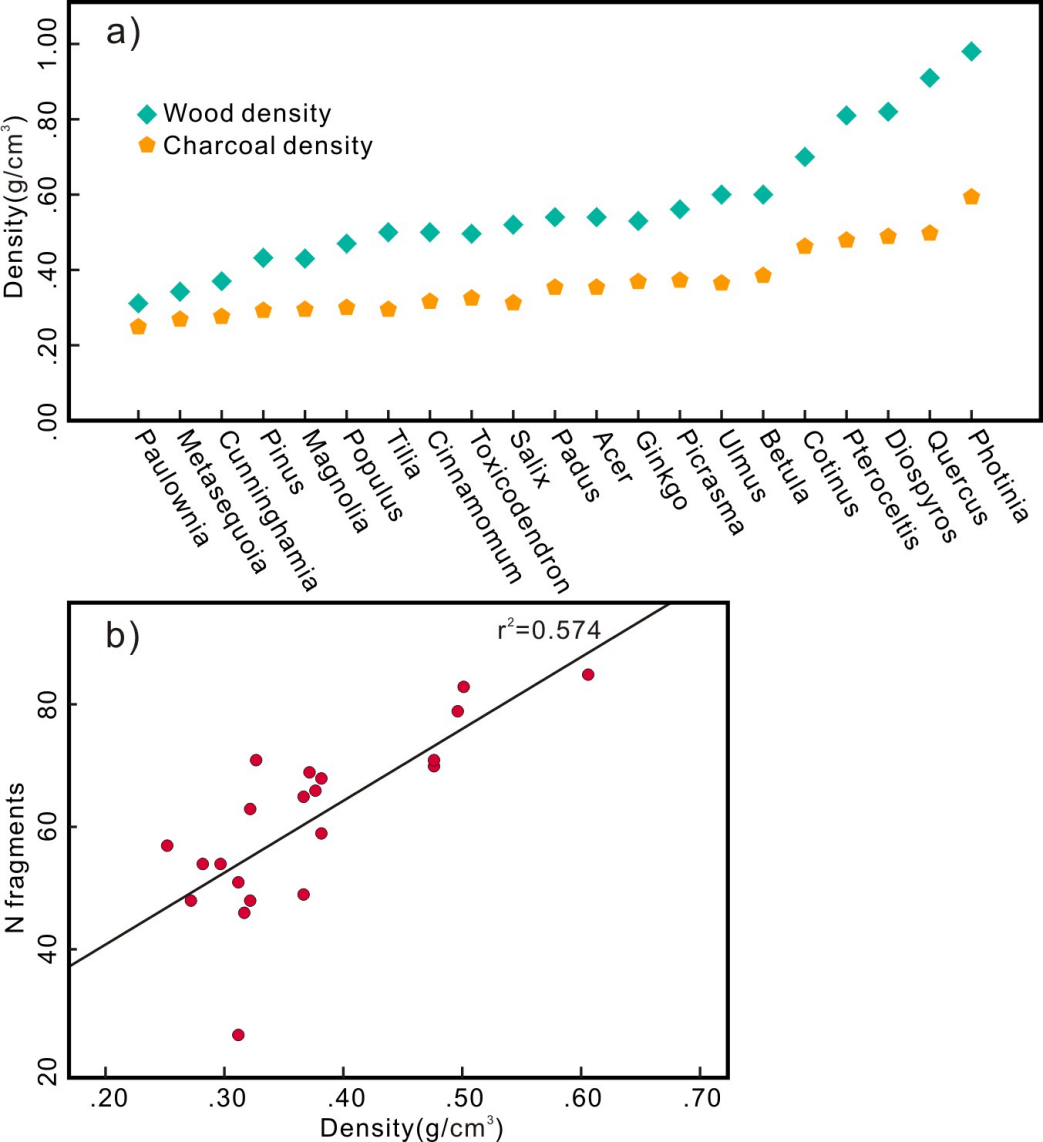
(a) Wood and charcoal density; (b) The relationship between charcoal density and number of fragments under wildfire conditions.

The physical properties of the different taxa are highly different, and the density and internal texture directly affect the mechanical properties of wood, which determine its compression resistance. During the process of wood carbonization, the density changes due to the volatilization of moisture and gas, but this change is consistent across the different taxa (Fig 6A). The densities of wood and charcoal exhibit a strong positive correlation [36]. Since the experiment under wildfire conditions is the closest to historic fire environment, we choose the number of fragments and density of the charcoal obtained in the wildfire experiment for correlation analysis. The results indicate that despite a low coefficient of determination (r^2^ = 0.574), there exists a significant positive correlation (r = 0.757) between the wood density (and therefore the charcoal density) and the total number of fragments (Fig 6B). Therefore, the taxa with a relatively high wood density tend to produce more fragments, and vice versa.

**Fig 7:**
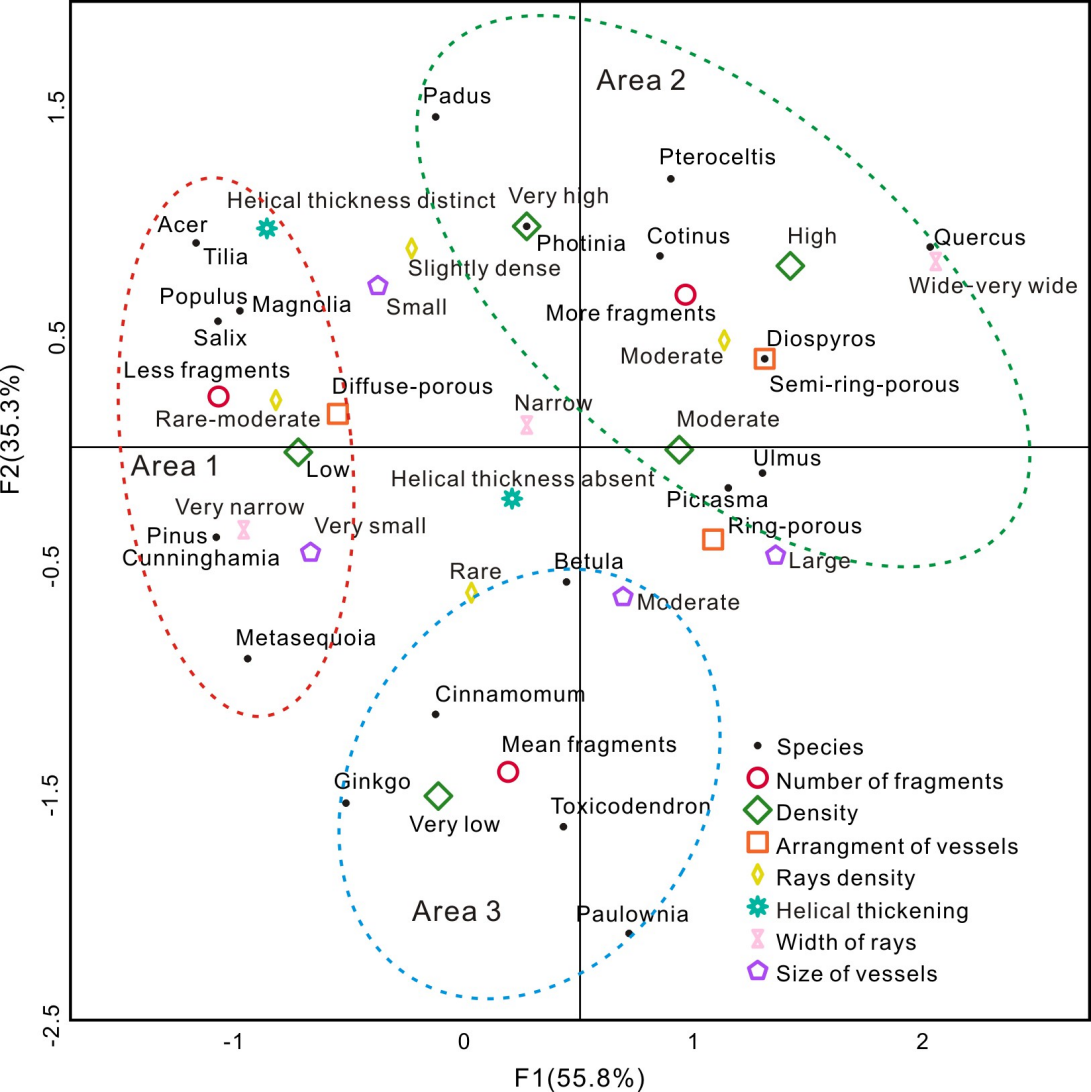
Multiple correspondence analysis (MCA).

To examine the influence of the wood texture on the crushing mode, multiple correspondence analysis (MCA) was performed on the main structural characteristics of wood, including the porosity, mean tangential diameter of the vessels, width of the rays, number of rays per millimeter and features of the helical thickening (Fig 7). In this study, the number of fragments with an average value within ±10% is defined as the median quantity that is moderately representative. The number of fragments more than 10% above the average value is defined as an excessive quantity that is overrepresentative, and the number of fragments more than 10% below the average value is defined as low quantity that is underrepresentative. In this paper, the classification of the wood air-dry density (very low/low/moderate/high/very high) is based on the wood physico-mechanical properties of the main tree taxa in China [47]. The classification of the wood ray width (very narrow/narrow/wide/very wide), ray density (rare/moderate/slightly dense/dense/very dense) and mean tangential diameter of the vessels (very small/small/moderate/large/very large) is based on Chinese Timber Annals [48]. The results indicate that the 21 taxa in this experiment are concentrated in three areas, namely, areas 1, 2, and 3 (Fig 7), containing taxa with few fragments, numerous fragments, and moderate fragments, respectively. The vast majority of the angiosperms contain vessels, but the size, arrangement, width and number of wood rays vary significantly among the different taxa.

The gymnosperms contain no vessels, resulting in tracheids accounting for more than 90% of the wood volume, tracheids mostly exhibit uniseriate rays (rarely two cells wide) and a relatively even internal texture. Therefore, the gymnosperms are mainly distributed in the fourth quadrant, and the angiosperms are distributed in all four quadrants, as shown in Fig 7.

Among the gymnosperms, *Pinus* (0.43 g/cm^3^), *Cunninghamia* (0.37 g/cm^3^) and *Metasequoia* (0.34 g/cm^3^) contain neatly arranged tracheids, exhibit relatively low densities, which are distributed in area 1, indicating underrepresentation. Compared to the other gymnosperms, the tracheids of *Ginkgo* (0.53 g/cm^3^) are different in size, arranged irregularly, relatively dense and thus are distributed in area 2, indicating moderate representation.

Among the angiosperms, diffuse-porous wood with a very low density (<0.55 g/cm^3^), very small to small vessels (<100 μm), and very narrow (1–2 cells) to narrow rays (3–4 cells) mainly occurs in area 1, indicating underrepresentation. Ring-porous/semi-ring-porous wood with a higher than moderate density (>0.55 g/cm^3^), larger than moderate vessels (>100 μm), and narrow (3–4 cells) to wide rays (>10 cells) or diffuse-porous wood with a very high density (>0.75 g/cm^3^), small to medium vessels (51–200 μm), and narrow rays (3–4 cells) is largely distributed in area 2, indicating overrepresentation. Ring-porous/semi-ring-porous wood with a very low density (<0.55 g/cm^3^), larger than moderate vessels (>100 μm), and narrow rays (3–4 cells) or diffuse-porous wood with a moderate density (0.55–0.65 g/cm^3^), small vessels (51–100 μm), and narrow rays (3–4 cells) primarily occurs in area 3, indicating suitable representation. In addition, the helical thickening increases the compressive resistance of charcoal [37]. The taxa with distinct helical thickening are all diffuse-porous wood, including *Acer*, *Magnolia*, *Tilia*, and *Padu*s. The first three taxa contain rare rays (>5/mm), which indicate underrepresentation, and *Padu*s contains slightly dense rays (10-13/mm) wood, which indicates overrepresented.

*Cinnamomum*, *Populus*, *Prunus* and *Salix* are all diffuse-porous wood taxa with a relatively low density. Among them, *Salix* and *Populus* contain small vessels (60–83 μm and 45–75 μm, respectively) and slightly dense uniseriate rays (9-11/mm and 9-14/mm, respectively), indicating underrepresentation. *Padu*s contains very small to small vessels (39–61 μm) and slightly dense multiseriate rays, indicating overrepresentation. *Cinnamomum* contains small to moderate vessels (70–145 μm) and rare-moderate multiseriate rays (4-8/mm), indicating moderate representation. Therefore, the width and density of rays may be important factors influencing charcoal fragmentation. Taxa with dense multiseriate rays tend to produce a relatively large number of charcoal fragments.

Generally, when the internal texture of charcoal exhibits large vessels or the vessel distribution is uneven, the charcoal tends to be subjected to uneven forces. As a result of the formation of weakened areas under stress, this charcoal breaks easily under compression and produces numerous fragments. Thus, compared to the diffuse-porous taxa, the ring-porous/semi-ring-porous taxa containing large vessels rather than small vessels, wide rays rather than narrow rays, dense rays rather than rare rays, and with helical thickening absence rather than helical thickening occurrence produce numerous fragments. Therefore, both the density and texture differences among the various charcoal taxa affect the degree of charcoal fragmentation.

The ring-porous/semi-ring-porous charcoal is fragile in terms of its texture. In our experiment, the influence of the density of the ring-porous/semi-ring-porous taxa on the degree of crushing is highlighted, indicating that the high-density taxa are overrepresented, while the low-density taxa are moderately represented. The diffuse-porous taxa and gymnosperms have relatively even textures, when compared to the ring-porous/semi-ring-porous taxa, they do not easily break. If diffuse-porous charcoal contains wide and dense rays, the uniformity of its texture is affected, and the charcoal is relatively fragile. Similarly, gymnosperms tend to produce more fragments when structural uniformity is poor. Therefore, in regard to ring-porous/semi-ring-porous wood with an uneven texture, the density affects the number of fragments produced in the charcoal. However, the degree of charcoal fragmentation of diffuse-porous wood and gymnosperms is related to their texture (Table 3).

**Table 3:** Relationship between wood/charcoal texture and representation.

Representation wood type	Ring-porous/semi-ring-porous trees	Diffuse-porous trees	Gymnosperms
Underrepresentation		Containing slightly dense uniseriate rays or rare multiseriate rays and with a distinct helical thickening; very low density (<0.55 g/cm^3^)	The tracheids are uniform with a regular arrangement
Moderate representation	Very low density (<0.55 g/cm^3^)	Containing rare to moderate multiseriate rays with helical thickening absence; low to moderate density (0.36–0.75 g/cm^3^)	The tracheids are not uniform and arranged irregularly
Overrepresentation	Higher than moderate density (>0.55 g/cm^3^)	Containing slightly dense multiseriate rays; higher than a low density (>0.36 g/cm^3^)	

In summary, the ring-porous/semi-ring-porous taxa with a higher than moderate density (>0.55 g/cm^3^) are overrepresented, while those with a very low to low density (<0.55 g/cm^3^) are moderately represented. The diffuse-porous taxa with slightly dense uniseriate rays, rare multiseriate rays and a distinct helical thickening are underrepresented, and those with slightly dense multiseriate rays are overrepresented, while those with rare to moderate multiseriate rays and helical thickening absence are moderately represented. Gymnosperms contain similar tracheids and uniseriate rays, and their texture is even. As such, they do not easily produce many fragments under stress. Therefore, they are usually underrepresented. Of these, *Ginkgo* is a broad-leaved taxa, and in contrast to the other conifers with neatly arranged tracheids, its tracheids are not uniform and arranged irregularly. Therefore, the number of fragments produced under compression is larger than that produced in the other conifers, indicating moderate representation.

### 4.3. From experimentation to the archeological context

*Acer*, *Betula*, *Pinus*, *Populus*, *Quercus*, *Salix* and *Ulmus* are the most common genera at the archeological sites in northern China. The preceding discussion has shown that the number of charcoal fragments produced by these taxa is quite different. Among them, *Quercus* and *Ulmus* tended to produce numerous fragments, and *Acer*, *Pinus*, *Populus* and *Salix* tended to produce relatively few fragments, while the number of fragments produced by *Betula* was close to the average value.

*Quercus* is not only a ubiquitous genus but is also an abundant genus; it is considered the dominant genus as well. However, because *Quercus* is a ringed porous wood, it contains large vessels, wide rays, and an uneven texture, which makes it susceptible to the generation of fragile zones, resulting in the ready formation of many fragments under compression. In addition, *Quercus* is relatively dense, and under the combined influence of the texture and density, it is overrepresented. The texture of *Ulmus* is similar to that of *Quercus*, and as it contains large vessels and wide rays and exhibits a moderate density, so it is also overrepresented.

As the most common gymnosperm at the archeological sites, *Pinus* is underrepresented due to its low density. *Acer*, *Betula*, *Populus* and *Salix* are all diffuse-porous wood taxa, with small vessels, fine rays and an even texture, similar to gymnosperms. Among them, *Salix* and *Populus* have a low density and contain slightly dense uniseriate rays, while *Acer* has a low density, rare multiseriate rays and a distinct helical thickening, and the above three taxa are underrepresented. *Betula* exhibits a moderate density, with rare to moderate multiseriate rays and without a helical thickening, indicating a moderate representation.

If the original vegetation amount is assumed to be 1 in this experiment and the number of fragments produced is x, the following representative calculation equation can be obtained:

$$\text{R}=\text{a}\left(1/\text{x}\right)$$ (Eq 1)

where a is the average number of fragments of charcoal at the various temperatures, and R is the representation coefficient of each taxa.

As most taxa attain similar R values at the different temperatures (S2 Table), the average value can be considered. The R values of the overrepresented *Quercus* and *Ulmus* are 0.74 and 0.88, respectively. The R values of the underrepresented *Acer*, *Pinus*, *Populus* and *Salix* are 1.20, 1.23, 1.17 and 1.23, respectively. When, in the future, reconstruction is performed via determination of the number of fragments at archeological sites, multiplication of the above taxa by their respective representation coefficient (R) value may yield better charcoal interpretation results.

## 5. Application example

A study of charcoal assemblages from the Huiduipo site in the southern Loess Plateau showed that *Quercus* (14.92%) accounted for the most, followed by *Fargesia* (13.37%), *Ulmus* (12.02%), *Sassafras* (9.88%), *Salix* (9.11%), and *Abies* (7.56%) (Table 4) [14]. According to the results of this paper, *Quercus* and *Ulmus* are overrepresented and *Salix* is underrepresented. While *Sassafras* and *Abies* were not present in the experiments of this paper, the internal texture and density of these two taxa could be compared with the experimental taxa [48], the results show that *Abies* has a uniform internal texture, low density and is similar to *Pinus* in physical properties, and it is therefore an underrepresented genus with a similar correction coefficient as *Pinus*; *Sassafras* is a ring-porous taxa, contains large vessels, wide rays and moderate density, and is similar to *Cotinus* in physical properties, and it is therefore an overrepresented taxa with a similar correction coefficient as *Cotinus*. Since the mechanical properties of bamboo charcoal are not studied in this paper, the representation of *Fargesia* is not discussed for now. After multiplying the above genera by the corresponding correction coefficients, the results show that *Salix* (11.20%) accounted for the most in the study area except for *Fargesia*, followed by *Quercus* (11.04%), *Ulmus* (10.57%), *Abies* (~9.07%) and *Sassafras* (~8.39%) (Table 4). The ranking of the major taxa in the study area changed after the correction, with *Salix* replacing *Quercus* as the dominant taxon.

**Table 4:** Genera correction and its growth environment from Huiduipo site.

Taxa	Original proportion (%)	Correction coefficients	Corrected proportion (%)	Growing environment
*Quercus*	14.92	0.74	11.04	Mostly grows on mountains and slopes at higher altitudes
*Ulmus*	12.02	0.88	10.57	Mostly grows on slopes and hill at lower altitudes
*Sassafras*	9.88	~0.85	8.39	Often distributes in plains and hilly areas at lower altitudes
*Salix*	9.11	1.23	11.20	Often distributes in plains and hilly areas along the river banks
*Abies*	7.56	~1.19	8.99	Mostly grows on mountains and slopes at higher altitudes

Modern plant ecological distribution patterns shows that *Quercus* is mostly distributed in higher altitude mountains and slopes; *Salix* is often distributed in plains and hilly areas along riverbanks. The Huiduipo site is located in the Guanzhong Plain at an altitude of approximately 400 meters, which is ecologically more suitable for the growth of *Salix*, thus, it became the first choice of our ancestors for fuelwood; this result is more in line with the "Principle of Least Effort" [49]. Quantitative paleoclimate reconstructions show a mean annual temperature of about 14.8°C and a mean annual precipitation of about 831mm in the study area during the 5600–6300 cal. yr BP, with a humid northern subtropical climate [14]. The proportion of subtropical taxa (*Sassafras*) in the corrected vegetation assemblage decreased and the proportion of temperate genera increased, which was more consistent with the local climate environment. Therefore, we believe that the corrected genera proportion can better reflect the local climatic environment and vegetation composition information.

## 6. Conclusion

The representativeness of charcoal fragments at archeological sites is investigated via the collection of modern wood samples for charring and compressing experiments. The following preliminary conclusions are obtained:

1. Carbonization causes inter-taxa differences in the number of charcoal fragments.
2. The density and texture of wood (charcoal) impose important influence on the number of charcoal fragments. The ring-porous/semi-ring-porous taxa with a higher than moderate density (>0.55 g/cm^3^) are overrepresented, while those with a very low to low density (<0.55 g/cm^3^) are moderately represented. The diffuse-porous genera containing slightly dense uniseriate rays, rare multiseriate rays and a distinct helical thickening are underrepresented, and those containing slightly dense multiseriate rays are overrepresented, while those containing rare to moderate multiseriate rays with helical thickening absence are moderately represented. Gymnosperm trees are generally underrepresented.
3. Among the ubiquitous genera at the archeological sites in northern China, *Quercus* and *Ulmus* may be overrepresented; *Acer*, *Pinus*, *Populus* and *Salix* may be underrepresented; and *Betula* may be moderately represented.

## Supporting information

S1 Table List of the species analysed with their weights, volumes and density changes, and the number of fragments of the 21 species and 4 heat treatments. Abbreviations: WD: wood density; T: temperature(°C); CD: charcoal density; CM: charcoal mass; CV: charcoal volume; NAC:Number of fragments after carbonization; >4 mm, 2–4 mm: number of fragments in each class size; Total: total number of fragments; Mean: Total(S1+S2)/2; NAF:Number of fragments after flotation.
(DOCX)

S2 Table The representative coefficient R-value of different temperature. (DOCX)

## References

[1] Antal MJ, Gronli M. The art, science, and technology of charcoal production. Ind Eng Chem Res. 2003; 42(8): 1619–1640.

[2] Schmid WI. Black carbon in soils and sediments: Analysis, distribution, implications, and current challenges. Global Biogeochem Cycles. 2000; 14(3): 777–793.

[3] Tan ZH, Huang CC, Pang JL, Zhou QY. Charcoal recorded Holocene fire history in the northern part of the Longdong Loess Plateau. Quatern Sci. 2008; 28(4): 733–738 (In Chinese).

[4] Cheng YM, Jiang XM, Li CS, Wang YF. Pliocene charcoals from Shanxi Province of China and their application to studies of prehistoric wildfires. Sci China. 2011; 54: 509–518.

[5] Zhao HL, Li XQ, Valerie AH. Holocene vegetation change in relation to fire and volcanic events in Jilin. Northeast China. Sci China: Earth Sci. 2015; 8: 1404–1419.

[6] Scott AC, Glasspool IJ. Observations and experiments on the origin and formation of inertinite group macerals. Int J Coal Geol. 2007; 70: 55–66.

[7] Scott AC. Charcoal recognition, taphonomy and uses in palaeoenvironmental analysis. Palaeogeogr Palaeoeocl. 2010; 291: 11–39.

[8] Chabal L. 1997. Forêtes et Sociétés en Languedoc (Nèolithique Final, antiquité Tardive). L’Anthracologie, Mèthode et Paéoécologie (Documents d’Archeologie Francaise 63). Éditions de la Maison des Sciences de l’Homme, Paris.

[9] Sun N, Li XQ. The quantitative reconstruction of the palaeoclimate between 5200 and 4300cal yr BP in the Tianshui basin, NW China. Clim Past. 2012; 8(2): 625–636.

[10] Sun N, Li XQ, Dodson J, Zhou XY, Zhao KL, Yang Q. Plant diversity of the Tianshui basin in the western Loess Plateau during the mid-Holocene charcoal records from archeological sites. Quatern Int. 2013; 308–309: 27–35.

[11] Wang SZ, Yue HB, Tang JG. High resolution reconstruction of ecological environment in Yin and Shang Dynasties. Relics from South. 2016; 2: 148–157 (In Chinese).

[12] Allué E, Picornell-Gelabert L, Daura J, Sanz M. Reconstruction of the palaeoenvironment and anthropogenic activity from the Upper Pleistocene/Holocene anthracological records of the NE Iberian Peninsula (Barcelona, Spain). Quatern Int, 2017; 457: 172–189.

[13] Sun N, Li XQ, Shang X, Zhou XY, Dodson J. Vegetation characteristics and palaeoclimate of Xiahe site in the Southern Loess Plateau during the mid-holocene based on fossil charcoal records. Quatern Sci. 2014; 34(1): 27–34 (In Chinese).

[14] Sun N, Li XQ, Dodson J, Zhou XY, Zhao KL, Yang Q. The quantitative reconstruction of temperature and precipitation in the Guanzhong Basin of the southern Loess Plateau between 6200 BP and 5600 BP. Holocene. 2016; 26(8): 1200–1207.

[15] Liu FW, Yang YS, Shi ZL, Storozum MJ, Dong GH. Human settlement and wood utilization along the mainstream of Heihe River basin, northwest China in historical period. Quatern Int. 2018; 516: 141–148.

[16] Sun N, Li XQ, Zhou XY, Zhao KL, Yang Q. Early smelting recorded by charcoal fossils in Hexi corridor, Gansu Province—An environment influence factor. Quatern Sci. 2010; 30: 319–325 (In Chinese).

[17] Bishop RR, Church MJ, Conwy PAR. Firewood, food and human niche construction: the potential role of Mesolithic hunter–gatherers in actively structuring Scotland’s woodlands. Quat Sci Rev. 2015; 108: 51–75.

[18] Marston JM. Modeling wood acquisition strategies from archaeological charcoal remains. J Archaeol Sci. 2009; 36: 2192–2200.

[19] Wang SZ, Wang ZL, He N. Study on the charcoal unearthed from Taosi site. Archaeology. 2011; 3: 91–93 (In Chinese).

[20] Wang SZ, Fang YM, Zhao ZJ. Vegetation paleoclimite and vegetation use during Longshan era: case studies of anthracology of Wadian site in Henan Province. Quatern Sci. 2012; 32: 226–235 (In Chinese).

[21] Wang SZ, Li H, Zhang RL. Tree exploitation and palaeo-environment at Heishuiguo Xichengyi site, Zhangye city, Gansu province revealed with excavated charcoal analysis. Quatern Sci. 2014; 34(1): 43–50 (In Chinese).

[22] Wang SZ, Yue HB, Tang JG, Yue ZW, He YL, Zhao ZJ. The utilization of trees in the middle and late Shang Dynasty—An analysis of the remains of trees unearthed from Shang City in Huanbei and Yin Ruins site. Relics from South. 2014; 0(3): 117–129 (In Chinese).

[23] Li XQ, Sun N, Dodson J, Zhou XY. Human activity and its impact on the landscape at the Xishanping site in the western Loess Plateau during 4800–4300 cal yr BP based on the fossil charcoal record. J Archaeol Sci. 2012; 39: 3141–3174.

[24] Li H, An CB, Dong WM, Wang SZ, Dong GH. The charcoal records of Qijia sites in Longdong basin and its significance. Quatern Sci. 2014; 34(1): 35–42 (In Chinese).

[25] Shen H, Li XQ, Zhou XY, Wu XH, Tang ZH, Sun N. Wood types and environment of the Tashkurgan region, Xinjiang, at 2500 cal yr BP, based on a record from the Ji’erzankale Necropolis. Rev Palaeobot Palyno. 2017; 238: 7–14.

[26] Shen H, Wu XH, Tang ZH, Zhou XY, Sun N, Li XQ. Wood usage and fire veneration in the Pamir, Xinjiang, 2500 yr BP. PLoS One. 2015; 10 (8): e01348472015. doi: 10.1371/journal.pone.0134847 26308646 PMC4550437

[27] Shen H, Zhou XY, Betts A, Jia PW, Zhao KL, Li XQ. Fruit collection and early evidence for horticulture in the Hexi Corridor, NW China, based on charcoal evidence. Veg Hist Archaeobot. 2018; 28(2): 187–197.

[28] Zhu XH, Li B, Ma CM, Zhu C, Wu L, Liu H. Late Neolithic phytolith and charcoal records of human activities and vegetation change in Shijiahe culture, Tanjialing site. PLoS One. 2017; 12(5): e017728. doi: 10.1371/journal.pone.0177287 28542219 PMC5438134

[29] Alonso MR, Zapata L, Díaz SP, Sáez JAL, Eraso J F. Selection of firewood in northern Iberia: Archaeobotanical data from three archaeological sites. Quatern Int. 2017; 431: 61–72.

[30] Kabukcu C. Woodland vegetation history and human impacts in south-central Anatolia 16000–6500cal BP: Anthracological results from five prehistoric sites in the Konya plain. Quat Sci Rev. 2017; 176: 85–100.

[31] Delhon C. Is choice acceptable? How the anthracological paradigm may hinder the consideration of fuel gathering as a cultural behaviour. Environ Archaeol. 2018; e1522783.

[32] Chabal L. L’etude palaeoecologique des sites protohistorique a partir des charbons de bois: la question de l’unite’ de meseure- denombrement des fragments ou pesees. 1988. In: Hackens T, Munaut AV, Till C (eds) Wood and archaeology. Acts of the European Symposium held at Louvain-la Neuve. Conseil de l’Europe, Strasbourg. 1987; 189–205.

[33] Théry-Parisot I, Chabal L, Chrzavzez J. Anthracology and taphonomy, from wood gathering to charcoal analysis. A review of the taphonomic processes modifying charcoal assemblages, in archaeological contexts. Palaeogeogr Palaeoeocl. 2010; 291: 142–153.

[34] Théry-Parisot I, Chabal L, M, Bouby L, Carré A. From wood to wood charcoal: an experimental approach to combustion/Du bois au charbons de bois, approche expérimentale de la combustion. 2010. In: Théry-Parisot I, Chabal L, Costamagno S (Eds). The Taphonomy of Burned Organic Residues and Combustion Features in Archaeological Contexts. Proceedings of the Round Table, Valbonne, May 27e29, 2008. Palethnologie 2: 81–93.

[35] Braadbaart F, Poole I Morphological. Chemical physical changes during charcoalification of wood and its relevance to archaeological contexts. J Archaeol Sci. 2008; 35: 2434–2445.

[36] Lancelotti C, Madella M, Ajithprasad P, Petrie CA. Temperature, compression and fragmentation: an experimental analysis to assess the impact of taphonomic processes on charcoal preservation. Archaeol Anthrop Sci. 2010; 2: 307–320.

[37] Chrzazvez J, Théry-Parisot I, Fiorucci J, Terral JF, Thibaut B. Impact of post-depositional processes on charcoal fragmentation and archaeobotanical implications: experimental approach combining charcoal analysis and biomechanics. J Archaeol Sci. 2014; 44: 30–42.

[38] An J H. 1992. The Chinese Archaeology. Shanghai Classic Publishing House, Shanghai.

[39] Swift LW, Elliott KJ, Ottmar RD, Vihnanek RE. Site preparation burning to improve southern Appalachian pine-hardwood stands: fifire characteristics and soil erosion, moisture, and temperature. Can J For Res. 1993; 23: 2242–2254.

[40] Costa LJ, Lima LVL, de Paula MO, Carneiro ADO, Reis MFD, Soares JD. Correlation between compression strength parallel of wood and charcoal of Eucalyptus clones. Sci For. 2018; 46(120): 606–613.

[41] Zhao ZJ. Field Work Method of plant archaeology-flotation method. Archaeology. 2004; 3: 80–87 (In Chinese).

[42] Yang HP, Yan R, Chen HP, Lee DH, Zheng CG. Characteristics of hemicellulose, cellulose and lignin pyrolysis. Fuel. 2007; 86: 1781–1788.

[43] Bartkowiak M, Zakrzewski R. Thermal degradation of lignins isolated from wood. J Therm Anal Calorim. 2004; 77(1): 295–304.

[44] Blankenhorn PR, Kline DE, Beall FC. Dynamic mechanical behavior of carbonized black cherry wood (Prunus serotina ehrh.). Carbon. 1973; 11(6): 603–611.

[45] Assis MR, Brancheriau L, Napoli A, Trugilho PF. Factors affecting the mechanics of carbonized wood: literature review. Wood Sci Technol. 2016; 50: 519–536.

[46] Blanchette RA. A review of microbial deterioration found in archaeological wood from different environments. Int Biodeter Biodegr. 2000; 46: 189–204.

[47] Institute of timber, Chinese Academy of Forestry Sciences. 1982. Wood physical and mechanical properties of main tree species in China. 1st ed. Beijing: China Forestry Press.

[48] Cheng JQ, Yang JJ, Liu P. 1992. Chinese Timber Annals. 1st ed. Beijing: China Forestry Publishing House.

[49] Shackleton CW, Prins F. Charcoal analysis and the principle of least effort—-A conceptual model. J Archaeol Sci, 1992, 19(6): 631–637.

