# Supplementary material for: Mechanical properties of charcoal and its representativeness of vegetation in northern China
Source: PLoS One. 2022 Apr 14;17(4):e0267044. doi: 10.1371/journal.pone.0267044 (PMC9009657; doi:10.1371/journal.pone.0267044)
Supplement: S1 Table — Abbreviations: WD: wood density; T: temperature(°C); CD: charcoal density; CM: charcoal mass; CV: charcoal volume; NAC:Number of fragments after carbonization; >4 mm, 2–4 mm: number of fragments in each class size; Total: total number of fragments; Mean: Total(S1+S2)/2; NAF:Number of fragments after flotation. (DOCX) [file pone.0267044.s001.docx]

**Table S1. List of the species analysed with their weights, volumes and density changes, and the number of fragments of the 21 species and 4 heat treatments. Abbreviations: WD: wood density; T: [temperature](https://fanyi.so.com/?src=onebox#temperature)(℃); CD: charcoal density; CM: charcoal mass; CV: charcoal volume; NAC:Number of fragments after carbonization; >4 mm, 2-4 mm: number of fragments in each class size; Total: total number of fragments; Mean: Total(S1+S2)/2; NAF:Number of fragments after flotation.**

| Species | WD/(g/cm^3^) | T/℃ | CD/(g/cm^3^) | | CV/cm^3^ | | CM/g | | **Charring experiment** | | | | **Compression experiment** | | | | | | | **Flotation experiment** | | |
| --- | --- | --- | --- | --- | --- | --- | --- | --- | --- | --- | --- | --- | --- | --- | --- | --- | --- | --- | --- | --- | --- | --- |
|  |  |  |  |  |  |  |  |  | Fissure | | NAC | | >4mm | | 2-4mm | | Total（>2mm） | | Mean（>2mm） | F | | Incrementrate（%） |
|  |  |  | S1 | S2 | S1 | S2 | S1 | S2 | S1 | S2 | S1 | S2 | S1 | S2 | S1 | S2 | S1 | S2 |  | S1 | S2 |  |
| Acer | 0.54 | 300 | 0.39 | 0.37 | 3.80 | 3.58 | 1.48 | 1.32 | 0 | 0 | 0 | 0 | 18 | 17 | 34 | 29 | 52 | 46 | 49 |  |  |  |
|  |  | 400 | 0.34 | 0.36 | 3.75 | 3.48 | 1.27 | 1.25 | 0 | 0 | 0 | 0 | 18 | 22 | 33 | 35 | 51 | 57 | 54 |  |  |  |
|  |  | 500 | 0.32 | 0.31 | 3.69 | 3.50 | 1.18 | 1.08 | 0 | 0 | 0 | 0 | 19 | 20 | 35 | 41 | 54 | 61 | 57 |  |  |  |
|  |  | WF | 0.36 | 0.38 | 4.12 | 4.04 | 1.48 | 1.53 | 0 | 0 | 0 | 0 | 19 | 17 | 34 | 30 | 53 | 47 | 50 | 55 | 50 | 5.0 |
| Betula | 0.60 | 300 | 0.43 | 0.40 | 4.23 | 4.32 | 1.81 | 1.72 | 0 | 0 | 0 | 0 | 19 | 20 | 41 | 38 | 60 | 58 | 59 |  |  |  |
|  |  | 400 | 0.38 | 0.39 | 3.52 | 3.44 | 1.33 | 1.37 | 1 | 1 | 2 | 0 | 19 | 22 | 43 | 47 | 62 | 69 | 66 |  |  |  |
|  |  | 500 | 0.35 | 0.37 | 3.20 | 3.22 | 1.12 | 1.19 | 0 | 1 | 0 | 2 | 22 | 21 | 43 | 49 | 65 | 70 | 68 |  |  |  |
|  |  | WF | 0.37 | 0.39 | 4.19 | 3.53 | 1.55 | 1.37 | 0 | 0 | 0 | 0 | 19 | 23 | 43 | 48 | 62 | 71 | 67 | 63 | 73 | 2.2 |
| Cinnamomum | 0.50 | 300 | 0.34 | 0.35 | 5.18 | 5.22 | 1.76 | 1.82 | 0 | 0 | 0 | 0 | 20 | 24 | 39 | 45 | 59 | 69 | 64 |  |  |  |
|  |  | 400 | 0.32 | 0.31 | 5.04 | 5.19 | 1.66 | 1.61 | 0 | 0 | 0 | 0 | 25 | 22 | 47 | 42 | 72 | 64 | 68 |  |  |  |
|  |  | 500 | 0.28 | 0.28 | 4.69 | 4.52 | 1.31 | 1.26 | 0 | 0 | 0 | 0 | 25 | 22 | 52 | 45 | 77 | 67 | 72 |  |  |  |
|  |  | WF | 0.33 | 0.32 | 5.61 | 6.04 | 1.85 | 1.79 | 0 | 2 | 0 | 0 | 22 | 21 | 45 | 39 | 67 | 60 | 64 | 72 | 65 | 3.7 |
| Cotinus | 0.70 | 300 | 0.48 | 0.50 | 5.04 | 5.11 | 2.41 | 2.56 | 0 | 0 | 0 | 0 | 21 | 25 | 44 | 50 | 65 | 75 | 70 |  |  |  |
|  |  | 400 | 0.45 | 0.47 | 4.62 | 5.04 | 2.10 | 2.37 | 0 | 0 | 0 | 0 | 28 | 26 | 51 | 45 | 79 | 71 | 75 |  |  |  |
|  |  | 500 | 0.40 | 0.44 | 4.89 | 4.95 | 1.98 | 2.18 | 2 | 2 | 0 | 2 | 26 | 27 | 51 | 53 | 77 | 80 | 79 |  |  |  |
|  |  | WF | 0.47 | 0.49 | 5.35 | 5.54 | 2.50 | 2.71 | 1 | 2 | 3 | 3 | 27 | 24 | 50 | 41 | 77 | 65 | 71 | 80 | 67 | 3.5 |
| Cunninghamia | 0.37 | 300 | 0.30 | 0.29 | 6.27 | 6.05 | 1.88 | 1.75 | 0 | 0 | 0 | 0 | 20 | 18 | 38 | 34 | 58 | 52 | 55 |  |  |  |
|  |  | 400 | 0.28 | 0.27 | 5.68 | 6.03 | 1.59 | 1.62 | 0 | 0 | 0 | 0 | 19 | 20 | 39 | 38 | 58 | 58 | 58 |  |  |  |
|  |  | 500 | 0.25 | 0.25 | 5.24 | 5.38 | 1.31 | 1.34 | 0 | 0 | 0 | 0 | 20 | 19 | 41 | 37 | 61 | 56 | 59 |  |  |  |
|  |  | WF | 0.29 | 0.28 | 5.96 | 5.81 | 1.72 | 1.62 | 1 | 1 | 0 | 0 | 17 | 19 | 35 | 39 | 52 | 58 | 55 | 54 | 60 | 3.6 |
| Diospyros | 0.82 | 300 | 0.53 | 0.54 | 4.48 | 4.35 | 2.37 | 2.34 | 0 | 0 | 0 | 0 | 28 | 25 | 54 | 47 | 82 | 72 | 77 |  |  |  |
|  |  | 400 | 0.48 | 0.47 | 4.26 | 4.33 | 2.04 | 2.03 | 1 | 2 | 3 | 3 | 26 | 28 | 51 | 60 | 77 | 88 | 83 |  |  |  |
|  |  | 500 | 0.44 | 0.45 | 4.14 | 4.16 | 1.82 | 1.87 | 2 | 2 | 2 | 3 | 24 | 28 | 54 | 60 | 78 | 88 | 83 |  |  |  |
|  |  | WF | 0.51 | 0.49 | 4.50 | 4.88 | 2.29 | 2.39 | 1 | 2 | 2 | 0 | 30 | 27 | 55 | 47 | 85 | 74 | 80 | 88 | 76 | 3.1 |
| Ginkgo | 0.53 | 300 | 0.38 | 0.41 | 3.99 | 4.15 | 1.51 | 1.70 | 0 | 0 | 0 | 0 | 20 | 24 | 34 | 40 | 54 | 64 | 59 |  |  |  |
|  |  | 400 | 0.35 | 0.38 | 4.02 | 4.10 | 1.40 | 1.55 | 0 | 0 | 0 | 0 | 23 | 21 | 43 | 41 | 66 | 62 | 64 |  |  |  |
|  |  | 500 | 0.34 | 0.32 | 3.74 | 3.58 | 1.27 | 1.14 | 1 | 2 | 2 | 0 | 25 | 23 | 50 | 44 | 75 | 67 | 71 |  |  |  |
|  |  | WF | 0.38 | 0.39 | 5.04 | 4.85 | 1.91 | 1.89 | 0 | 2 | 0 | 0 | 24 | 20 | 44 | 32 | 68 | 52 | 60 | 71 | 53 | 3.3 |
| Magnolia | 0.43 | 300 | 300 | 0.31 | 0.3 | 4.23 | 4.01 | 1.31 | 0 | 0 | 0 | 0 | 11 | 10 | 20 | 17 | 31 | 27 | 29 |  |  |  |
|  |  | 400 | 0.29 | 0.29 | 3.16 | 3.28 | 0.91 | 0.95 | 2 | 1 | 2 | 2 | 11 | 10 | 20 | 19 | 30 | 29 | 30 |  |  |  |
|  |  | 500 | 0.28 | 0.26 | 3.20 | 3.03 | 0.89 | 0.90 | 2 | 2 | 3 | 2 | 12 | 11 | 24 | 21 | 36 | 32 | 34 |  |  |  |
|  |  | WF | 0.31 | 0.32 | 4.12 | 4.25 | 1.27 | 1.36 | 0 | 1 | 0 | 0 | 9 | 10 | 17 | 18 | 26 | 28 | 27 | 29 | 28 | 3.6 |
| Metasequoia | 0.34 | 300 | 0.30 | 0.29 | 5.04 | 4.57 | 1.52 | 1.32 | 0 | 0 | 0 | 0 | 19 | 18 | 33 | 31 | 52 | 49 | 51 |  |  |  |
|  |  | 400 | 0.26 | 0.26 | 4.23 | 4.20 | 1.09 | 1.09 | 1 | 0 | 0 | 0 | 19 | 17 | 37 | 34 | 56 | 51 | 54 |  |  |  |
|  |  | 500 | 0.24 | 0.25 | 4.11 | 3.94 | 0.98 | 0.98 | 1 | 0 | 0 | 0 | 19 | 18 | 40 | 38 | 59 | 56 | 58 |  |  |  |
|  |  | WF | 0.28 | 0.27 | 4.86 | 4.67 | 1.36 | 1.26 | 2 | 1 | 0 | 0 | 19 | 16 | 34 | 28 | 53 | 44 | 49 | 55 | 45 | 3.0 |
| Padus | 0.54 | 300 | 0.37 | 0.40 | 3.56 | 3.71 | 1.34 | 1.48 | 0 | 0 | 0 | 0 | 23 | 22 | 50 | 44 | 73 | 66 | 70 |  |  |  |
|  |  | 400 | 0.37 | 0.34 | 3.54 | 3.45 | 1.31 | 1.19 | 0 | 0 | 0 | 0 | 24 | 23 | 51 | 49 | 75 | 72 | 74 |  |  |  |
|  |  | 500 | 0.29 | 0.32 | 3.31 | 3.40 | 0.95 | 1.10 | 2 | 2 | 0 | 2 | 22 | 26 | 47 | 50 | 69 | 76 | 73 |  |  |  |
|  |  | WF | 0.36 | 0.38 | 4.12 | 4.08 | 1.48 | 1.55 | 1 | 1 | 2 | 2 | 25 | 21 | 44 | 41 | 69 | 62 | 66 | 72 | 63 | 3.0 |
| Paulownia | 0.31 | 300 | 0.27 | 0.29 | 4.75 | 5.03 | 1.28 | 1.45 | 0 | 0 | 0 | 0 | 19 | 21 | 38 | 44 | 57 | 66 | 62 |  |  |  |
|  |  | 400 | 0.24 | 0.25 | 4.23 | 4.44 | 1.01 | 1.10 | 0 | 0 | 0 | 0 | 18 | 22 | 37 | 45 | 55 | 67 | 61 |  |  |  |
|  |  | 500 | 0.20 | 0.23 | 3.56 | 3.75 | 0.71 | 0.86 | 0 | 0 | 0 | 0 | 20 | 19 | 43 | 37 | 63 | 56 | 60 |  |  |  |
|  |  | WF | 0.27 | 0.24 | 4.82 | 4.61 | 1.30 | 1.11 | 1 | 2 | 3 | 3 | 18 | 19 | 37 | 42 | 55 | 61 | 58 | 57 | 62 | 2.5 |
| Photinia | 0.98 | 300 | 0.64 | 0.68 | 3.74 | 3.50 | 2.39 | 2.40 | 0 | 0 | 0 | 0 | 23 | 21 | 43 | 39 | 48 | 60 | 54 |  |  |  |
|  |  | 400 | 0.54 | 0.59 | 3.44 | 3.46 | 1.85 | 2.04 | 1 | 2 | 2 | 2 | 23 | 22 | 49 | 42 | 72 | 64 | 68 |  |  |  |
|  |  | 500 | 0.52 | 0.56 | 3.30 | 3.46 | 1.71 | 1.93 | 2 | 2 | 0 | 2 | 23 | 24 | 51 | 55 | 74 | 80 | 77 |  |  |  |
|  |  | WF | 0.62 | 0.60 | 4.12 | 3.77 | 2.55 | 2.26 | 1 | 3 | 3 | 3 | 27 | 26 | 62 | 57 | 89 | 83 | 86 | 93 | 87 | 4.6 |
| Picrasma | 0.56 | 300 | 0.40 | 0.43 | 3.97 | 4.53 | 1.79 | 1.96 | 0 | 0 | 0 | 0 | 21 | 23 | 40 | 48 | 61 | 71 | 66 |  |  |  |
|  |  | 400 | 0.37 | 0.35 | 4.69 | 4.23 | 1.73 | 1.48 | 0 | 0 | 0 | 0 | 22 | 24 | 47 | 49 | 69 | 73 | 71 |  |  |  |
|  |  | 500 | 0.34 | 0.32 | 4.42 | 4.24 | 1.51 | 1.35 | 0 | 0 | 0 | 0 | 25 | 23 | 51 | 47 | 78 | 70 | 74 |  |  |  |
|  |  | WF | 0.39 | 0.38 | 4.36 | 4.62 | 1.83 | 1.85 | 0 | 0 | 0 | 0 | 23 | 21 | 49 | 44 | 72 | 65 | 69 | 75 | 66 | 2.9 |
| Pinus | 0.43 | 300 | 0.34 | 0.31 | 5.14 | 5.43 | 1.74 | 1.68 | 0 | 0 | 0 | 0 | 18 | 17 | 29 | 32 | 47 | 49 | 48 |  |  |  |
|  |  | 400 | 0.27 | 0.28 | 4.83 | 4.93 | 1.34 | 1.38 | 0 | 0 | 0 | 0 | 17 | 19 | 34 | 39 | 51 | 58 | 55 |  |  |  |
|  |  | 500 | 0.25 | 0.26 | 4.83 | 4.93 | 1.34 | 1.33 | 0 | 0 | 0 | 0 | 20 | 21 | 36 | 39 | 56 | 60 | 58 |  |  |  |
|  |  | WF | 0.33 | 0.30 | 5.08 | 5.24 | 1.67 | 1.57 | 0 | 0 | 0 | 0 | 17 | 16 | 32 | 38 | 49 | 54 | 52 | 51 | 55 | 2.9 |
| Populus | 0.47 | 300 | 0.33 | 0.32 | 4.23 | 4.42 | 1.40 | 1.41 | 0 | 0 | 0 | 0 | 17 | 16 | 39 | 32 | 56 | 48 | 52 |  |  |  |
|  |  | 400 | 0.29 | 0.27 | 3.59 | 3.40 | 1.05 | 0.90 | 0 | 0 | 0 | 0 | 18 | 21 | 35 | 44 | 53 | 65 | 59 |  |  |  |
|  |  | 500 | 0.26 | 0.28 | 3.28 | 3.32 | 0.86 | 0.93 | 0 | 1 | 0 | 2 | 20 | 19 | 39 | 34 | 59 | 53 | 56 |  |  |  |
|  |  | WF | 0.31 | 0.34 | 4.61 | 4.53 | 1.42 | 1.55 | 0 | 1 | 0 | 0 | 17 | 16 | 35 | 30 | 52 | 46 | 49 | 54 | 47 | 3.0 |
| Pteroceltis | 0.81 | 300 | 0.52 | 0.51 | 4.91 | 4.89 | 2.55 | 2.49 | 0 | 0 | 0 | 0 | 25 | 25 | 50 | 41 | 75 | 66 | 71 |  |  |  |
|  |  | 400 | 0.49 | 0.48 | 4.57 | 4.52 | 2.23 | 2.16 | 0 | 0 | 0 | 0 | 25 | 27 | 47 | 52 | 72 | 79 | 76 |  |  |  |
|  |  | 500 | 0.44 | 0.43 | 4.53 | 4.25 | 1.99 | 1.94 | 1 | 1 | 0 | 0 | 23 | 25 | 56 | 50 | 79 | 75 | 77 |  |  |  |
|  |  | WF | 0.48 | 0.48 | 4.90 | 5.01 | 2.35 | 2.39 | 0 | 0 | 0 | 0 | 23 | 24 | 45 | 51 | 68 | 75 | 72 | 69 | 76 | 1.3 |
| Quercus | 0.91 | 300 | 0.52 | 0.53 | 4.23 | 4.11 | 2.19 | 2.17 | 0 | 0 | 0 | 0 | 31 | 30 | 54 | 49 | 85 | 79 | 82 |  |  |  |
|  |  | 400 | 0.48 | 0.50 | 4.12 | 4.35 | 1.97 | 2.17 | 0 | 0 | 0 | 0 | 28 | 32 | 54 | 60 | 82 | 92 | 87 |  |  |  |
|  |  | 500 | 0.48 | 0.46 | 4.06 | 4.20 | 1.94 | 1.93 | 2 | 2 | 0 | 0 | 29 | 32 | 54 | 61 | 83 | 93 | 88 |  |  |  |
|  |  | WF | 0.49 | 0.52 | 4.46 | 4.38 | 2.18 | 2.27 | 0 | 0 | 0 | 0 | 25 | 32 | 54 | 57 | 79 | 89 | 84 | 81 | 92 | 2.9 |
| Salix | 0.52 | 300 | 0.34 | 0.36 | 4.48 | 4.58 | 1.52 | 1.64 | 0 | 0 | 0 | 0 | 16 | 17 | 30 | 28 | 47 | 45 | 46 |  |  |  |
|  |  | 400 | 0.33 | 0.32 | 3.94 | 3.77 | 1.30 | 1.19 | 0 | 0 | 0 | 0 | 19 | 19 | 33 | 31 | 52 | 50 | 51 |  |  |  |
|  |  | 500 | 0.26 | 0.29 | 3.85 | 3.78 | 1.01 | 1.09 | 3 | 2 | 2 | 0 | 17 | 19 | 31 | 39 | 48 | 58 | 53 |  |  |  |
|  |  | WF | 0.31 | 0.29 | 4.36 | 4.41 | 1.35 | 1.27 | 1 | 1 | 3 | 4 | 18 | 22 | 34 | 36 | 52 | 58 | 55 | 55 | 60 | 4.5 |
| Tilia | 0.50 | 300 | 0.33 | 0.31 | 4.23 | 4.89 | 1.39 | 1.51 | 0 | 0 | 0 | 0 | 17 | 17 | 34 | 36 | 51 | 53 | 52 |  |  |  |
|  |  | 400 | 0.28 | 0.29 | 4.48 | 3.96 | 1.25 | 1.14 | 0 | 0 | 0 | 0 | 18 | 15 | 38 | 35 | 56 | 50 | 53 |  |  |  |
|  |  | 500 | 0.26 | 0.25 | 3.93 | 4.37 | 1.02 | 1.09 | 0 | 0 | 0 | 0 | 20 | 19 | 42 | 38 | 62 | 57 | 60 |  |  |  |
|  |  | WF | 0.32 | 0.32 | 5.06 | 4.85 | 1.61 | 1.55 | 2 | 1 | 2 | 3 | 17 | 15 | 32 | 29 | 49 | 44 | 47 | 50 | 44 | 1.0 |
| Toxicodendron | 0.50 | 300 | 0.34 | 0.35 | 4.54 | 4.17 | 1.54 | 1.45 | 0 | 0 | 0 | 0 | 23 | 22 | 39 | 38 | 62 | 60 | 61 |  |  |  |
|  |  | 400 | 0.33 | 0.33 | 3.99 | 4.04 | 1.31 | 1.33 | 0 | 0 | 0 | 0 | 23 | 24 | 46 | 41 | 69 | 65 | 67 |  |  |  |
|  |  | 500 | 0.30 | 0.29 | 4.12 | 3.81 | 1.23 | 1.10 | 0 | 0 | 0 | 0 | 22 | 22 | 45 | 48 | 67 | 70 | 69 |  |  |  |
|  |  | WF | 0.34 | 0.32 | 4.86 | 4.42 | 1.65 | 1.41 | 0 | 0 | 0 | 0 | 25 | 22 | 50 | 46 | 75 | 68 | 72 | 78 | 70 | 3.4 |
| Ulmus | 0.60 | 300 | 0.41 | 0.40 | 4.47 | 4.62 | 1.70 | 1.83 | 0 | 0 | 0 | 0 | 23 | 22 | 48 | 45 | 71 | 67 | 69 |  |  |  |
|  |  | 400 | 0.36 | 0.38 | 4.46 | 4.21 | 1.61 | 1.59 | 0 | 1 | 0 | 0 | 25 | 21 | 52 | 47 | 77 | 68 | 73 |  |  |  |
|  |  | 500 | 0.32 | 0.30 | 4.31 | 4.45 | 1.38 | 1.51 | 2 | 2 | 2 | 2 | 24 | 25 | 45 | 53 | 69 | 78 | 74 |  |  |  |
|  |  | WF | 0.38 | 0.37 | 4.23 | 4.56 | 1.60 | 1.68 | 1 | 2 | 2 | 0 | 22 | 24 | 42 | 51 | 64 | 75 | 70 | 66 | 78 | 3.5 |
