# Supplementary material for: Mechanical properties of charcoal and its representativeness of vegetation in northern China
Source: PLoS One. 2022 Apr 14;17(4):e0267044. doi: 10.1371/journal.pone.0267044 (PMC9009657; doi:10.1371/journal.pone.0267044)
Supplement: S2 Table — (DOCX) [file pone.0267044.s002.docx]

**Table S2. The representative coefficient R-value of different temperature.**

| Species  R  T/℃ | 300(a=57) | 400(a=63) | 500(a=69) | WT(a=62) | Mean |
| --- | --- | --- | --- | --- | --- |
| Acer | 1.2 | 1.19 | 1.18 | 1.24 | 1.2 |
| Betula | 1 | 0.97 | 0.99 | 0.93 | 0.97 |
| Cinnamomum | 0.92 | 0.94 | 0.93 | 0.97 | 0.94 |
| Cotinus | 0.84 | 0.85 | 0.85 | 0.87 | 0.85 |
| Cunninghamia | 1.07 | 1.1 | 1.14 | 1.13 | 1.11 |
| Diospyros | 0.77 | 0.77 | 0.81 | 0.78 | 0.78 |
| Ginkgo | 1 | 1 | 0.94 | 1.03 | 0.99 |
| Magnolia | 2.03 | 2.13 | 1.97 | 2.3 | 2.11 |
| Metasequoia | 1.16 | 1.19 | 1.16 | 1.27 | 1.19 |
| Padus | 0.84 | 0.86 | 0.92 | 0.94 | 0.89 |
| Paulownia | 0.95 | 1.05 | 1.12 | 1.07 | 1.05 |
| Photinia | 1.09 | 0.94 | 0.87 | 0.72 | 0.91 |
| Picrasma | 0.89 | 0.9 | 0.91 | 0.9 | 0.9 |
| Pinus | 1.23 | 1.16 | 1.16 | 1.19 | 1.19 |
| Populus | 1.13 | 1.08 | 1.2 | 1.27 | 1.17 |
| Pteroceltis | 0.83 | 0.84 | 0.87 | 0.86 | 0.85 |
| Quercus | 0.72 | 0.74 | 0.76 | 0.74 | 0.74 |
| Salix | 1.28 | 1.25 | 1.26 | 1.13 | 1.23 |
| Tilia | 1.13 | 1.21 | 1.12 | 1.32 | 1.19 |
| Toxicodendron | 0.97 | 0.96 | 0.97 | 0.86 | 0.94 |
| Ulmus | 0.86 | 0.88 | 0.91 | 0.89 | 0.88 |
