# Supplementary material for: Mechanical properties of charcoal and its representativeness of vegetation in northern China
Source: PLoS One. 2022 Apr 14;17(4):e0267044. doi: 10.1371/journal.pone.0267044 (PMC9009657; doi:10.1371/journal.pone.0267044)
Supplement: Attachment — Submitted filename: Response to Reviewers.docx [file pone.0267044.s003.docx]

Dear Editor:

Thank you for arranging a timely review for our manuscript. We appreciate the comments from the reviewers that contribute to our manuscript greatly.

We have carefully evaluated the comments and suggestions, and respond to these point by point below. All changes made to the Revised Manuscript with Track Changes text are shown in red.

Best wishes.

Sincerely,

Nan Sun and Xiabo Li

Reviewer #1: There are minor matters with the English.

Thank you, the language was re-edited by professional agency.

The authors should note they are analysing woody genera yet the text refers to species. I suggest the word species be replaced by genera or taxa as appropriate.

Thank you for your suggestion, the word “species” has been replaced by “taxa” in the revised manuscript.

Reviewer #2: The restoration of paleovegetation through charcoal analysis is important to understand ancient environment and human activity. This paper used simulation experiments to discuss the fragmentation patterns among different tree species, and this will help more accurately restore paleovegetation. The experiment design is reasonable and the results is meaningful. One more advice is as follows:
The authors should use known charcoal data from one site with known environment, and then apply their equation to the data to observe whether their restoration is consistent with the known environment.

Thank you for your suggestion, we have added one part to apply our experiment result. Please see L 352-383.
